# Psychosis in children of separated parents: A systematic review and meta-analysis

**DOI:** 10.1192/j.eurpsy.2019.15

**Published:** 2020-01-31

**Authors:** Luis Ayerbe, María Pérez-Piñar, Quintí Foguet-Boreu, Salma Ayis

**Affiliations:** 1 Department of Public Health and Primary Care, University of Cambridge, Cambridge, United Kingdom; 2 Centre of Primary Care and Public Health, Queen Mary University of London, London, United Kingdom; 3 Carnarvon Medical Centre, Southend-on-Sea, United Kingdom; 4 Department of Psychiatry, Vic University Hospital, Vic, Spain; 5 Faculty of Health Sciences and Welfare, University of Vic-Central University of Catalonia (UVic-UCC), Vic, Spain; 6 School of Population Health and Environmental Sciences, King’s College London, London, United Kingdom; 7 National Institute for Health Research Biomedical Research Centre, Guy’s and St Thomas’ NHS Foundation Trust, London, United Kingdom; 8 National Institute for Health Research Collaboration for Leadership in Applied Health Research and Care, South London at King’s College Hospital NHS Foundation Trust, London, United Kingdom

**Keywords:** childhood adversities, divorce, parental separation, psychotic disorders, schizophrenia

## Abstract

**Background.:**

Parental separation is a very common childhood adversity. The association between other adverse childhood experiences and an increased risk of psychosis has been reported. However, the evidence on the risk of psychosis for children of separated parents is limited. In this systematic review, cohort, case–control, and cross-sectional studies, comparing the risk of psychotic disorders for people with and without separated parents, were searched, critically appraised, and summarized.

**Methods.:**

Studies were searched in PubMed, EMBASE, PsycINFO, and the Web of Science, from database inception to September 2019. A meta-analysis, using random-effects models, was undertaken to obtain pooled estimates of the risk of psychosis among participants with separated parents.

**Results.:**

Twelve studies, with 305,652 participants from 22 countries, were included in the review. A significantly increased risk of psychosis for those with separated parents was observed, with a pooled odds ratio: 1.53 (95% confidence interval [CI]: 1.29–1.76), *p* < 0.001. The association remained significant when cohort, case–control, and cross-sectional studies were analyzed separately. The five cohort studies included in this review showed and increased risk of psychosis with odds ratio: 1.47 (95% CI: 1.26–1.69), *p* < 0.001.

**Conclusions.:**

Parental separation is a common childhood adversity associated with an increased risk of psychosis. Although the risk for an individual child of separated parents is still low, given the high proportion of couple that separate, the increased rates of psychosis may be substantial in the population. Further studies on the risk of psychosis in those with separated parents, and the explanatory factors for this association, are required.

## Introduction

The dissolution of marriage is common, affecting 46% of married couples in the USA, 42% in Australia, and 43% in Europe [1–3]. In many cases, separated couples have children together (i.e., 47% of couples in Australia, 57% in Spain, 48% in the UK, with an average of 1.8 and 2 children per separated couple in Australia or the UK, respectively) [2,4,5].

Parental separation has consequences in children’s life, including exposure to conflict between parents, economic loss, reduced contact with one parent, and increased life stressors such as changing schools, childcare, or homes. Parental separation has become one of the most common adverse childhood experiences, second only to socioeconomic disadvantage [6,7]. Two systematic reviews have reported that children of separated parents have an increased risk of mood and drug use disorders [8,9]. Evidence reported in two other systematic reviews also shows an increased risk of psychosis in people who have experienced other adversities during childhood such as physical or sexual abuse [10,11]. However, the studies on the associations between parental separation and psychosis have not been critically appraised or summarized. Therefore, the relevance of parents’ separation on the development of psychotic disorders remains uncertain for clinicians, patients and parents, social workers, psychologists, counselors, and educators.

Strong evidence on the association between parental separation and psychosis could identify people at higher risk of having psychotic disorders and inform a proactive and multidisciplinary approach to them. This systematic review and meta-analysis present an up-to-date critical appraisal and summary of the available evidence on the risk of developing psychotic disorders in children of separated parents.

## Methods

The Meta-analysis of Observational Studies in Epidemiology criteria were used to undertake this review [12]. Observational studies, including published papers and conference abstracts, reporting risk of psychotic disorders in children of separated couples were searched in PUBMED, EMBASE, PsychINFO, and Web of Science from database inception until the 7th of September 2019. The following search strategy was used: (((((((((((“Divorce”[Mesh]) OR ((((((parent*) OR Family) OR Marital) OR “Marriage”[Mesh])) AND separation)) OR ((((“Marriage”[Mesh]) OR Marital)) AND dissolution))))))))) AND (((((((psychoses) OR psychosis) OR “Hallucinations”[Mesh]) OR “Delusions”[Mesh]) OR “Schizophrenia”[Mesh]) OR (“Schizophrenia Spectrum and Other Psychotic Disorders”[Mesh])) OR “Psychotic Disorders”[Mesh])).

The bibliography lists of all papers fitting the inclusion criteria, and relevant reviews identified in the initial search, were checked for further articles. Papers citing all the included studies or relevant reviews were also searched in the Web of Science and considered for inclusion. There were no restrictions on the basis of language, sample size, or duration of follow-up.

The following articles were not included: interventional studies; papers that presented only associations with specific domains or outcomes of psychosis; studies with composite outcome (i.e., mental health disorders) unless separate results for psychosis were presented; those reporting outcome as a continuous variable (i.e., score in psychosis scales); articles presenting only subclinical psychosis or drug induced psychotic disorders; studies comparing participants with different mental health disorders instead of those with and without psychosis; papers in which the exposure was separation between parent and child but parental separation was not one of the reasons for this; those conducted in specific patient subpopulations (i.e., exposure assessed only on participants receiving specific medication); studies reporting results only from univariate analysis; and ecological studies.

One researcher (L.A.) conducted all searches of eligible studies. Eligibility was confirmed by two other researchers (M.P.-P. and S.A.). In some cases, the authors of the studies were contacted for any clarifications required including possible publication of two papers from the same sample. Where data from the same participants were presented in more than one paper, only data from the study with larger sample size were included in the analysis. Two researchers extracted the data from all the eligible studies (L.A. and S.A.) using a standardized data-collection form. The risk of bias and overall methodological quality of the studies fitting the inclusion criteria was assessed using the Quality Assessment Tools for Case–Control, Cohort and Cross-Sectional Studies of the National Institute of Health (USA) [13].

### Statistical methods

Pooled estimates of odds ratios (ORs) were obtained using random-effects models [14]. ORs and 95% confidence intervals (CI) were used to present results. *p* value ≤0.05 was used as a criterion for significance. The heterogeneity between studies was measured using I-squared index (*I* ^2^), which represents inconsistency of the studies included and estimates the percentage of the total variation due to heterogeneity rather than chance [15] All studies included had reported ORs as an estimate of the association between parental separation and psychosis, except two where the relative risk (RR) [16] and the hazard ratio (HR) [17] were used. The two estimates were treated as proxies for ORs. For the RR, the prevalence of psychosis was low (<10%), making the two estimates fairly similar; for the HR, the estimate is widely used as a proxy for OR [18,19]. To assess heterogeneity and its impact on the pooled estimates, sensitivity analyses were used: (1) stratifying studies by study design (case–control, cohort, and cross-sectional), (2) excluding studies where the exposure was the separation between parent and child for different reasons, including, but not exclusively, parental separation, (3) excluding cross sectional studies, and (4) excluding studies that had a very wide CI or reported results very different to the rest. A funnel plot was used to investigate possible publication bias. The software Stata v14.0 was used for the analysis [20].

## Results

**Figure 1.**
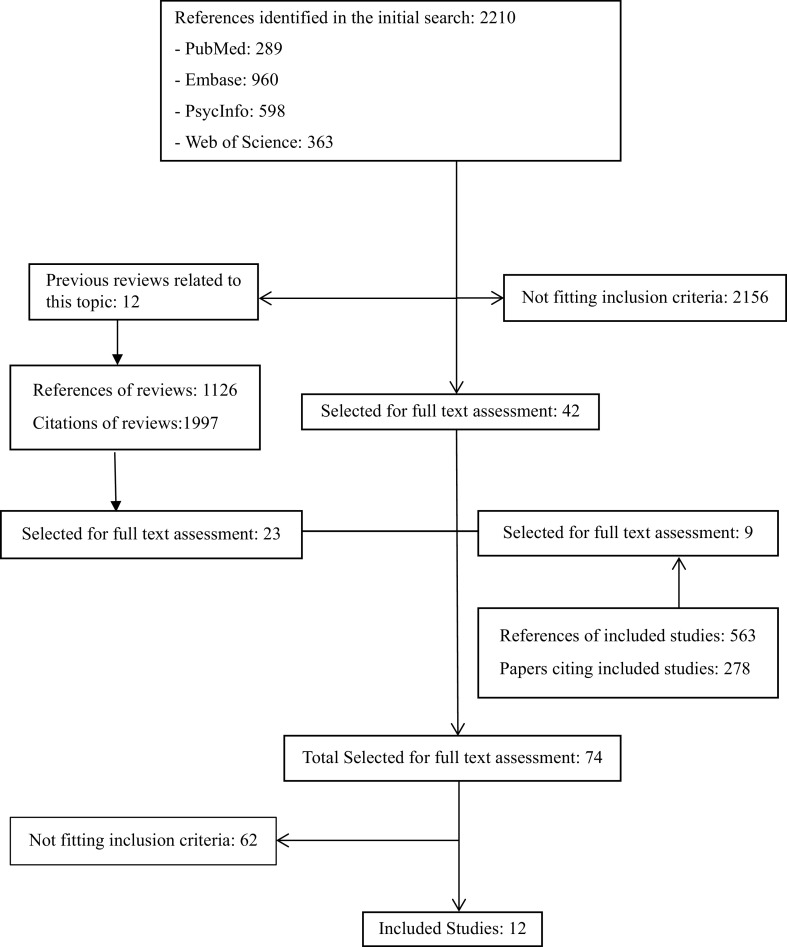
Results of literature search.

The electronic search produced 2,210 references, 289 from PUBMED, 960 from EMBASE, 598 from PsychInfo, and 363 from Web of Science. Twelve reviews relevant to the topic, with a total of 1,126 references, and cited by 1,997 papers were also identified [10,11,21–30]. The results of the search are presented in Figure 1.

Twelve papers including in total 305,652 participants were considered eligible [16,17,31–40]. These studies had been conducted in 22 different countries, 9 of them were based in Europe, 2 in Asia, and 1 collected data from participants based in America, Africa, Asia, and Europe. Six were case–control studies [31–35,37]. Five were cohort studies with follow up between 3 and 26 years [16,17,38–40]. One of them only included male participants [16]. The remainder study had a cross-sectional design and data have been collected on psychosis and parental separation at an earlier time [36]. The sample size ranged from 347 to 107,704 participants. In seven studies, the exposure was exclusively parental separation [16,17,33,35,36,38,39]. The other five papers used a broader exposure representing the child becoming separated from one or both parents due to parental separation or other causes such as death of parent or abandonment of child [31,32,34,37,40]. Four studies used a clinical diagnosis of schizophrenia as outcome [16,31,33,38]. Seven studies used a clinical diagnosis of psychotic disorders [17,32,34,35,37,39,40]. Finally, one study used psychotic disorder as outcome defined with a score above a cut-off point in a scale [36]. The characteristics of all included papers are presented in Table 1. They were all considered to be of high quality (Appendix A).

**Table 1.** Characteristics of the studies included in the meta-analysis.

Author, year, country	Design	Follow-up	*N*	Exposure (*N*)	Outcome measure (*n*)	Risk
Furukawa, 1998 Japan	CC	–	347	Parental separation or death at age < 16 (113)	Schizophrenia DSMIII-R (225)	Men OR Dad loss: 0.84 (0.25–2.86) Mum loss: 0.44 (0.12–1.62) Women OR Dad loss: 1.23 (0.50–3.01) Mum loss: 1.61 (0.52–4.96)
Hansagi, 2000 Sweden	Cohort	19 years	47,033	Parental separation at age < 21 (4,914)	Schizophrenia ICD8 (295)	RR: 1.3 (0.9–1.8)
Morgan, 2007 UK	CC	–	781	Parental separation or child abandonment at age < 16 (240)	Psychosis ICD10 (390)	OR: 2.45 (1.66–3.59)
Rubino, 2009 Italy	CC	–	483	Parental separation at age < 17 (173)	Schizophrenia DSM 4 (173)	OR: 5.54 (2.23–13.74)
Stilo, 2013 UK	CC	–	504	Parental separation or child abandonment at age < 17 (165)	Psychosis ICD10 (278)	OR: 1.84 (1.21–2.80)
Trotta, 2015 UK	CC	–	541	Parental separation at age < 17 (248)	Psychosis ICD10 (285)	OR: 1.96 (1.322–.91), *p* = 0.001
Bjorkenstam, 2016 Sweden	Cohort	24 years	10,7704	Parental separation at age 3–14 (23,265)	Psychosis ICD10 (400)	HR: 1.8 (1.5–2.2)
Shevlin, 2016 Denmark	Cohort	22 years	58,690	Parental separation or death at age < 10 (16,908)	Psychosis aged 10–21 ICD10 (389)	OR: 1.44 (1.14–1.81)
Stilo, 2017 UK	CC	–	634	Parental separation or child abandonment at age < 17 (281)	Psychosis ICD10 (332)	OR: 2.43 (1.64–3.58)
McGrath, 2017 Belgium, Brazil, China, Iraq, Colombia, USA, France, Peru, Germany, Italy, Lebanon, Mexico, Netherlands, Nigeria, Portugal, Romania, Spain	CS		23,998	Parental separation at age < 18 (1,320)	Psychosis CIDI (1,661)	OR: 1.5 (1.2–1.9)
Merikukka, 2018 Finland	Cohort	26 years	58,739	Parental separation at age < 16 (13,294)	Schizophrenia ICD10 (229)	OR: 1.28 (0.95–1.71)
Zhang, 2019 China	Cohort	3 years	6,198	Parental separation at age < 18	Psychosis MINI	OR: 8.35 (0.40–175.02)

Abbreviations: CIDI, Composite International Diagnostic Interview; CC, case–control study; CS, cross-sectional study; DSM, Diagnostic and Statistical Manual of mental disorders; ICD, International Classification of Diseases; MINI, Mini-International Neuropsychiatric Interview.

**Figure 2.**
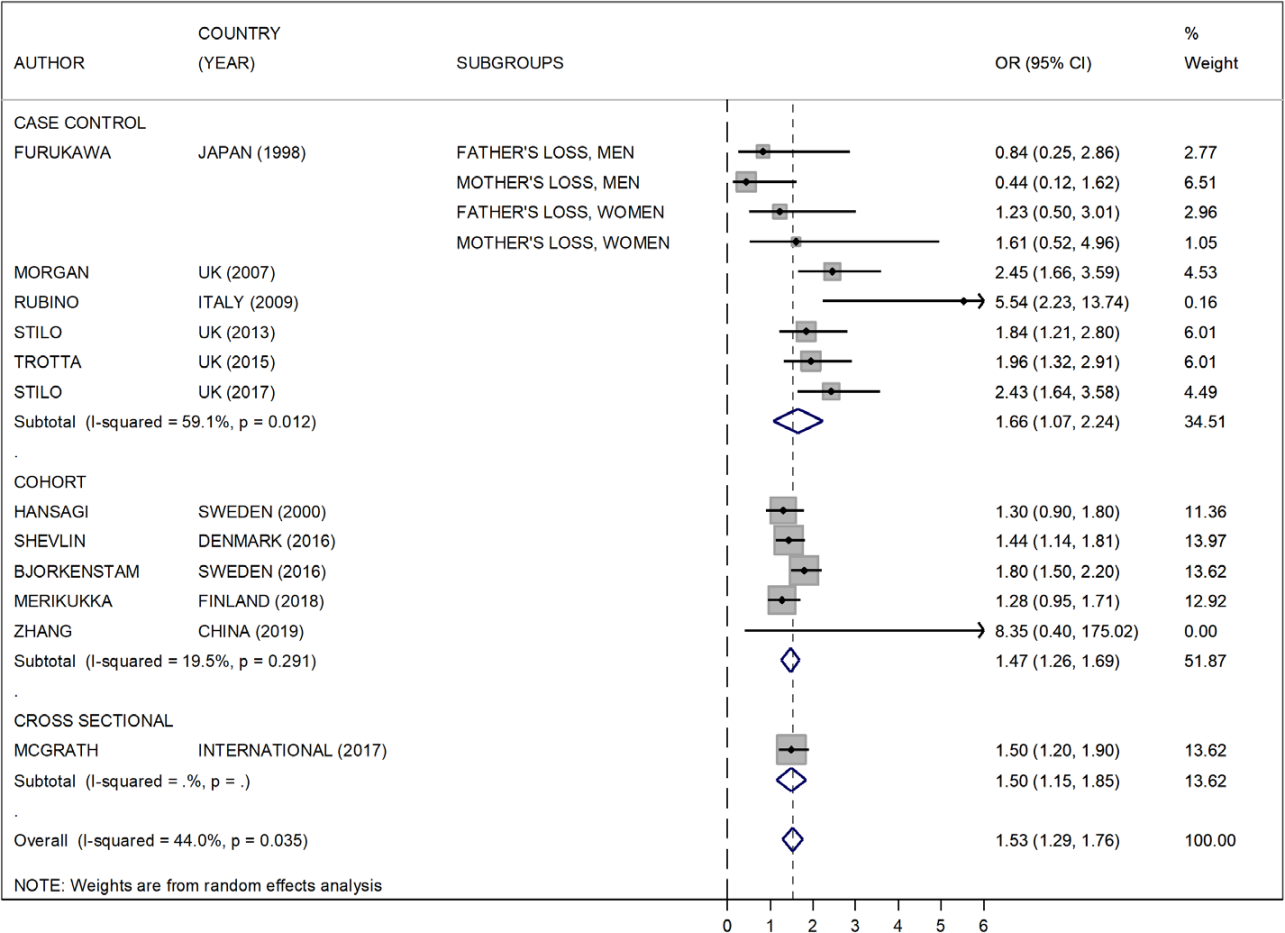
Risk of psychosis in children of separated parents (all studies included).

**Figure 3.**
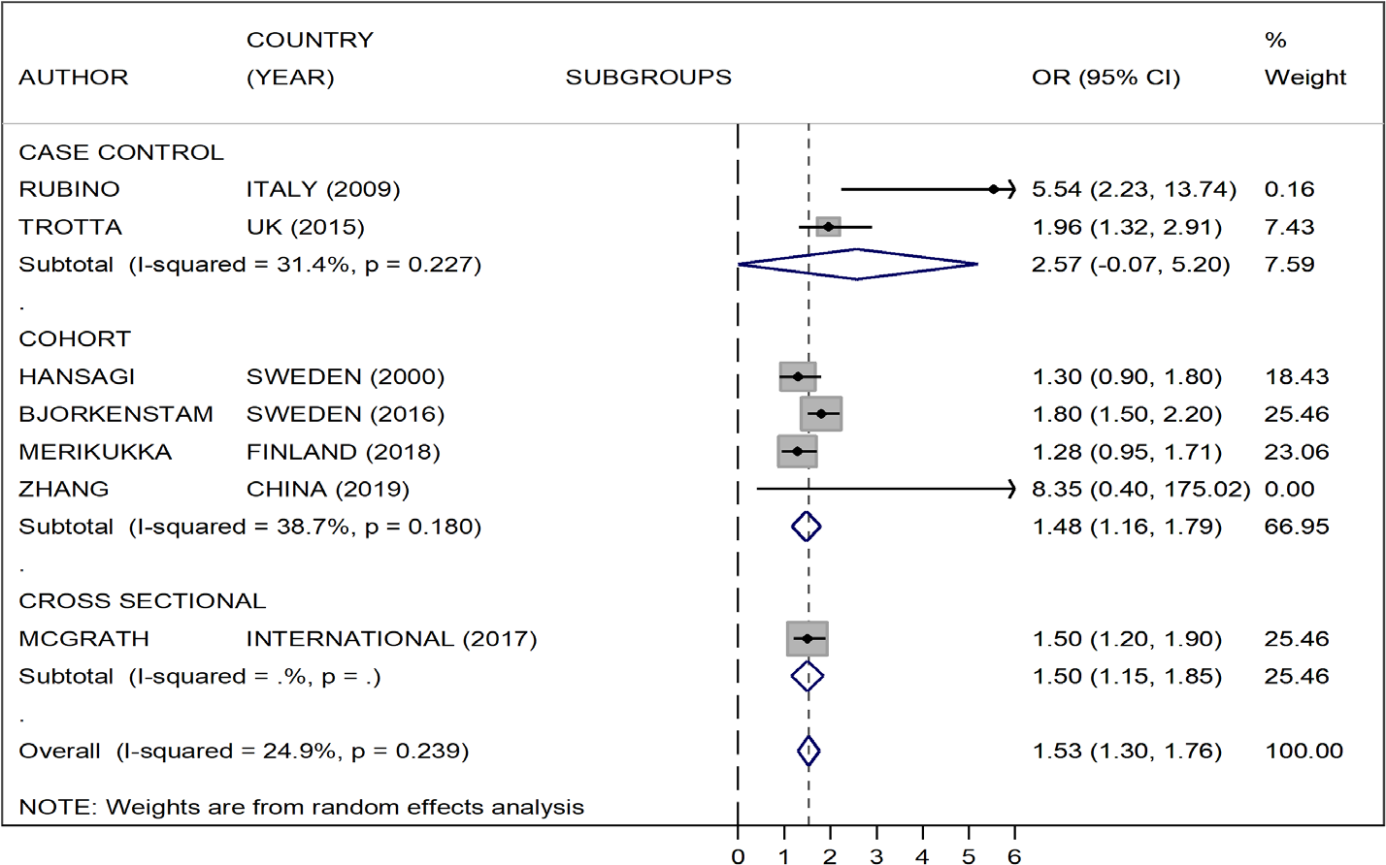
Risk of psychosis in children of separated parents excluding studies where the exposure was the separation between parent and child for different reasons, including, but not exclusively, parental separation.

The meta-analysis showed that parental separation is significantly associated with psychosis, OR: 1.53 (1.29–1.76; Figure 2). The heterogeneity of the studies was 44.0% and varied depending on the study design. The association was also significant when studies were categorized by design, cohort, case–control, or cross-sectional studies for each individual category. The five cohort studies showed and increased risk of psychosis, OR: 1.47 (1.26–1.69), consistent with the overall pooled estimate and a lower level of heterogeneity (19.5%). When the studies where the exposure was the separation between parent and child for different reasons, including, but not exclusively, parental separation, were excluded from the meta-analysis, the overall risk of psychosis was OR: 1.53 (1.30–1.76) with heterogeneity 24.9% (Figure 3). When the cross-sectional study was excluded, the overall risk of psychosis changes minimally, OR: 1.54 (1.26–1.81), with heterogeneity of 48.0% (Appendix B).

One cohort study had a very wide CI. The study weight approached zero with minimal impact on the overall estimate [39]. One case–control study reported a risk between two and five times higher than the one reported in all the other studies [33]. However, when these two studies were removed from the meta-analysis the pooled OR did not show major changes: 1.52 (1.29, 1.76) and heterogeneity 48.0%. All pooled estimates showed significantly increased risk with *p* < 0.001. The funnel plot demonstrated a reasonable symmetry suggesting that publication bias and other sources of biases due to methodology, quality, and small studies effect are unlikely (Appendix C).

## Discussion

Children of separated parents have a risk of psychosis increased by 53%. This association remains significant when studies are categorized by methodology or design. Two previous systematic reviews have reported the association between parental separation and other mental health problems with an increased risk of depression and cannabis dependence for children of separated parents [8,9]. However, to the best of our knowledge, this is the first review reporting on the association between parental separation and psychotic disorders.

The increased risk of psychosis for children of separated parents is consistent with the increased risk for those exposed to other childhood adversities, such as sexual, emotional, physical abuse, or neglect, reported in another systematic review [10].

The mechanisms that explain the association between parental separation and psychotic disorders are likely to be complex and include neurological, psychological, and social factors. A prolonged situation of stress in a child, experienced before or after the separation, can lead to some pathophysiological changes including dysregulation of the hypothalamic–pituitary–adrenal axis and increased oxidative stress in the central nervous system, which are both associated with the development of psychosis [26,41]. Childhood adversities can also have a psychological impact on mood, stress regulation, self-concept, social roles, cognitive styles, coping responses, attachment representations, and dissociative mechanisms [11,26,28]. Family arrangements after separation can also have an explanatory role in the association between parental separation and psychotic disorders. A large cohort study reported that children growing up with single parents had an increased risk of serious psychiatric disorders, suicide, or alcohol addiction [42]. The increased rate of substance use in children of separated parents could also be an explanatory factor for the association between parental separation and psychotic disorders [9,43,44]. However, the evidence on the neurological, psychological, and social mechanisms that link parental separation and psychotic disorders requires further research. For example, the effect of many familial factors is unclear; the role of family dysfunctionality, domestic violence, drug, or alcohol abuse in parents, together with physical or mental illness in the family, the presence of siblings, and financial issues require more research. Future studies could also address the vulnerability of patients of certain age and gender, those who experience other adversities in childhood or adult life, together with genetic factors and medical conditions that may increase the risk of mental illness. The protective factors that make resilient some people exposed to childhood adversities including parental separation also need more studies [26].

This review has some limitations. The diversity of the methods across studies may have an effect on the external validity of each individual one. In general, case–control studies have several limitations including that the temporality between cause and effect is more difficult to establish, there can be selection bias of the controls, and the information collected from the control is prone to recall bias. Case–controlled studies showed a significant association between parental separation and psychosis, but larger heterogeneity than cohort studies. Furthermore, only one doctor conducted the initial search (L.A.). Even so, all data were checked for accuracy on multiple occasions and all analyses were conducted several times and checked by a senior statistician (S.A.). Finally, some publications may have been miscoded or missed altogether.

This study has strengths as well. The comprehensive search and critical assessment of studies conducted in this review allows estimation of the association between parental separation and psychosis obtained on a large number of participants assessed worldwide. The use of a random effect model, based on the assumption that studies were independently conducted and do not necessarily share a common effect size, allowing for more uncertainty of the final summary estimate, was a conservative choice. The overall estimate remained significant despite the increased width of the confidence intervals, providing support to the significance of the findings. The reasonably symmetrical funnel plot suggests provides no evidence of publication bias or small study effects [45].

The risk of psychosis is higher for children exposed to other adversities such as sexual, emotional, physical abuse, or neglect (OR > 2) than for children of separated parents. However, the higher frequency of parental separation can result in a larger number of cases of psychosis associated with it in the population [1–3]. Parents, educators, and clinicians have to be alert to the symptoms that could indicate the need for intervention [46]. Usually, the intervention of mental health professionals is not necessary after a separation, although this adversity as well as others should be routinely recorded, when seeing patients with potential psychosis. The approach to children of separated parents may need to be multidisciplinary as the associations between parental separation and other adverse outcomes such as poorer physical health, academic performance, and social interaction have also been reported [22,47].

## Conclusion

Parental separation is associated with an increased risk of psychosis. This should be acknowledged by all professionals working with children of separated parents. Although the risk for an individual child of separated parents is still very low, given the high proportion of couple that separate, the increased rates of psychosis may be substantial in the population. Future studies addressing the possible mechanisms for this association are required.

## Appendix A

### Assessment of case‑control studies.

**Table:**

	Study and year					
	Furukawa 1998	Morgan 2007	Rubino 2009	Stilo 2013	Trotta 2015	Stilo 2017
1. Was the research question or objective in this paper clearly stated and appropriate?	Yes	Yes	Yes	Yes	Yes	Yes
2. Was the study population clearly specified and defined?	Yes	Yes	Yes	Yes	yes	Yes
3. Did the authors include a sample size justification?	Yes	Yes	Yes	Yes	Yes	Yes
4. Were controls selected or recruited from the same or similar population that gave rise to the cases (including the same timeframe)?	Yes	Yes	Yes	Yes	Yes	Yes
5. Were the definitions, inclusion and exclusion criteria, algorithms or processes used to identify or select cases and controls valid, reliable, and implemented consistently across all study participants?	Yes	Yes	Yes	Yes	Yes	Yes
6. Were the cases clearly defined and differentiated from controls?	Yes	Yes	Yes	Yes	Yes	Yes
7. If less than 100 percent of eligible cases and/or controls were selected for the study, were the cases and/or controls randomly selected from those eligible?	No	Yes	No	No	No	Yes
8. Was there use of concurrent controls?	Yes	Yes	Yes	Yes	Yes	Yes
9. Were the investigators able to confirm that the exposure/risk occurred prior to the development of the condition or event that defined a participant as a case?	Yes	Yes	Yes	Yes	Yes	Yes
10. Were the measures of exposure/risk clearly defined, valid, reliable, and implemented consistently (including the same time period) across all study participants?	Yes	Yes	Yes	Yes	Yes	Yes
11. Were the assessors of exposure/risk blinded to the case or control status of participants?	No	No	No	No	No	No
12. Were key potential confounding variables measured and adjusted statistically in the analyses? If matching was used, did the investigators account for matching during study analysis?	Yes	Yes	Yes	Yes	Yes	Yes

### Assessment of cohort and cross sectional studies.

**Table:**

	Study and year					
	Hansagi 2000	Bjorkenstam 2016	Shevlin 2016	McGrath 2017	Merikukka 2018	Zhang 2019
1. Was the research question or objective in this paper clearly stated?	Yes	Yes	Yes	Yes	Yes	Yes
2. Was the study population clearly specified and defined?	Yes	Yes	Yes	Yes	Yes	Yes
3. Was the participation rate of eligible persons at least 50%?	Yes	Yes	Yes	Yes	Yes	Yes
4. Were all the subjects selected or recruited from the same or similar populations (including the same time period)? Were inclusion and exclusion criteria for being in the study prespecified and applied uniformly to all participants?	Yes	Yes	Yes	Yes	Yes	Yes
5. Was a sample size justification, power description, or variance and effect estimates provided?	Yes	Yes	Yes	Yes	Yes	Yes
6. For the analyses in this paper, were the exposure(s) of interest measured prior to the outcome(s) being measured?	Yes	Yes	Yes	No	Yes	Yes
7. Was the timeframe sufficient so that one could reasonably expect to see an association between exposure and outcome if it existed?	Yes	Yes	Yes	Yes	Yes	Yes
8. For exposures that can vary in amount or level, did the study examine different levels of the exposure as related to the outcome (e.g., categories of exposure, or exposure measured as continuous variable)?	NA	NA	NA	NA	NA	NA
9. Were the exposure measures (independent variables) clearly defined, valid, reliable, and implemented consistently across all study participants?	Yes	Yes	Yes	Yes	Yes	Yes
10. Was the exposure(s) assessed more than once over time?	No	No	No	No	No	Yes
11. Were the outcome measures (dependent variables) clearly defined, valid, reliable, and implemented consistently across all study participants?	Yes	Yes	Yes	Yes	Yes	Yes
12. Were the outcome assessors blinded to the exposure status of participants?	No	No	No	No	No	No
13. Was loss to follow-up after baseline 20% or less?	Yes	Yes	Yes	Yes	Yes	Yes
14. Were key potential confounding variables measured and adjusted statistically for their impact on the relationship between exposure(s) and outcome(s)?	Yes	Yes	Yes	Yes	Yes	Yes

## References

[1] National Centre for Health Statistics. Marriage and divorce. https://www.cdc.gov/nchs/fastats/marriage-divorce.htm [accessed September 2019].

[2] Australian Bureau of Statistics. Marriages and divorces. http://www.abs.gov.au/ausstats/abs@.nsf/mf/3310.0 [accessed September 2019].

[3] Eurostat. Divorce indicators. https://ec.europa.eu/eurostat/web/population-demography-migration-projections/data/database [accessed August 2019].

[4] Instituto Nacional de Estadistica. Estadística de Nulidades, Separaciones y Divorcios. https://www.ine.es/prensa/ensd_2016.pdf [accessed September 2019].

[5] Office for National Statistics. Divorces in England and Wales, children of divorced couples: historical data. https://www.ons.gov.uk/peoplepopulationandcommunity/birthsdeathsandmarriages/divorce/datasets/divorcesinenglandandwaleschildrenofdivorcedcouples [accessed August 2019].

[6] Sacks V , Murphey D . The prevalence of adverse childhood experiences, nationally, by estate, and by race or ethnicity. https://www.childtrends.org/publications/prevalence-adverse-childhood-experiences-nationally-state-race-ethnicity [accessed July 2019].

[7] Department of Health and Social Care. Routine enquiry about childhood adversity. https://www.gov.uk/government/publications/routine-enquiry-about-adverse-childhood-experiences-implementation-pack-evaluation [accessed July 2019].

[8] Sands A , Thompson EJ , Gaysina D . Long-term influences of parental divorce on offspring affective disorders: a systematic review and meta-analysis. J Affect Disord. 2017;218:105–114. 28463711 10.1016/j.jad.2017.04.015

[9] Schlossarek S , Kempkensteffen J , Reimer J , Verthein U . Psychosocial determinants of cannabis dependence: a systematic review of the literature. Eur Addict Res. 2016;22(3):131–144. 26551358 10.1159/000441777

[10] Varese F , Smeets F , Drukker M , Lieverse R , Lataster T , Viechtbauer W , et al. Childhood adversities increase the risk of psychosis: a meta-analysis of patient-control, prospective- and cross-sectional cohort studies. Schizophr Bull. 2012;38(4):661–671. 22461484 10.1093/schbul/sbs050 PMC3406538

[11] Matheson SL , Shepherd AM , Pinchbeck RM , Laurens KR , Carr VJ . Childhood adversity in schizophrenia: a systematic meta-analysis. Psychol Med. 2013;43(2):225–238. 22716913 10.1017/S0033291712000785

[12] Stroup DF , Berlin JA , Morton SC , Olkin I , Williamson GD , Rennie D , et al. Meta-analysis of observational studies in epidemiology: a proposal for reporting. Meta-analysis Of Observational Studies in Epidemiology (MOOSE) group. JAMA. 2000;283:2008–2012. 10789670 10.1001/jama.283.15.2008

[13] Quality assessment tool for observational cohort and cross-sectional studies. National Institute of Health. https://www.nhlbi.nih.gov/health-pro/guidelines/in-develop/cardiovascular-risk-reduction/tools/cohort [accessed September 2019].

[14] DerSimonian R , Kacker R . Random-effects model for meta-analysis of clinical trials: an update. Contemp Clin Trial. 2007;28(2):105–114. 10.1016/j.cct.2006.04.004 16807131

[15] Higgins J , Thompson SG . Quantifying heterogeneity in a meta‐analysis. Stat Med. 2002;21(11):1539–1558. 12111919 10.1002/sim.1186

[16] Hansagi H , Lena B , Sven A . Parental divorce: psychosocial well-being, mental health and mortality during youth and young adulthood. Eur J Public Health. 2000;10:86–92.

[17] Bjorkenstam E , Burstrom B , Vinnerljung B , Kosidou K . Childhood adversity and psychiatric disorder in young adulthood: An analysis of 107,704 Swedes. J Psychiatr Res. 2016;77:67–75. 26994339 10.1016/j.jpsychires.2016.02.018

[18] Zhang J , Yu KF . What's the relative risk? A method of correcting the odds ratio in cohort studies of common outcomes. JAMA. 1998;280(19):1690–1691. 9832001 10.1001/jama.280.19.1690

[19] Stare J , Maucort-Boulch D . Odds ratio, hazard ratio and relative risk. Metodoloski Zvezki. 2016;13(1):59.

[20] StataCorp. Stata statistical software: release 14. College Station, TX: StataCorp LP, 2015.

[21] Olin SC , Mednick SA . Risk factors of psychosis: identifying vulnerable populations premorbidly. Schizophr Bull. 1996;22(2):223–240. 8782283 10.1093/schbul/22.2.223

[22] Amato PR . Children of divorce in the 1990s: an update of the Amato and Keith (1991) meta-analysis. J Fam Psychol. 2001;15(3):355–370. 11584788 10.1037//0893-3200.15.3.355

[23] De Sousa P , Varese F , Sellwood W , Bentall RP . Parental communication and psychosis: a meta-analysis. Schizophr Bull. 2014;40(4):756–768. 23800431 10.1093/schbul/sbt088 PMC4059429

[24] Fusar-Poli P , Tantardini M , De Simone S , Ramella-Cravaro V , Oliver D , Kingdon J , et al. Deconstructing vulnerability for psychosis: Meta-analysis of environmental risk factors for psychosis in subjects at ultra high-risk. Eur Psychiatry. 2017;40:65–75. 27992836 10.1016/j.eurpsy.2016.09.003

[25] Morgan C , Fisher H . Environment and schizophrenia: environmental factors in schizophrenia: childhood trauma—a critical review. Schizophr Bull. 2007;33(1):3–10. 17105965 10.1093/schbul/sbl053 PMC2632300

[26] Morgan C , Gayer-Anderson C . Childhood adversities and psychosis: evidence, challenges, implications. World Psychiatry. 2016;15(2):93–102. 27265690 10.1002/wps.20330 PMC4911761

[27] Trotta A , Murray RM , Fisher HL . The impact of childhood adversity on the persistence of psychotic symptoms: a systematic review and meta-analysis. Psychol Med. 2015;45(12):2481–2498. 25903153 10.1017/S0033291715000574

[28] Bentall RP , de Sousa P , Varese F , Wickham S , Sitko K , Haarmans M , et al. From adversity to psychosis: pathways and mechanisms from specific adversities to specific symptoms. Soc Psychiatry Psychiatr Epidemiol. 2014;49(7):1011–1022. 24919446 10.1007/s00127-014-0914-0

[29] East L , Jackson D , O'Brien L . Father absence and adolescent development: a review of the literature. J Child Health Care. 2006;10(4):283–295. 17101621 10.1177/1367493506067869

[30] Peh OH , Rapisarda A , Lee J . Childhood adversities in people at ultra-high risk (UHR) for psychosis: a systematic review and meta-analysis. Psychol Med. 2019;1–13. 10.1017/S003329171800394X 30616701

[31] Furukawa T , Mizukawa R , Hirai T , Fujihara S , Kitamura T , Takahashi K . Childhood parental loss and schizophrenia: evidence against pathogenic but for some pathoplastic effects. Psychiatry Res. 1998;81(3):353–362. 9925186 10.1016/s0165-1781(98)00113-9

[32] Morgan C , Kirkbride J , Leff J , Craig T , Hutchinson G , McKenzie K , et al. Parental separation, loss and psychosis in different ethnic groups: a case-control study. Psychol Med. 2007;37(4):495–503. 17094816 10.1017/S0033291706009330

[33] Rubino IA , Nanni RC , Pozzi DM , Siracusano A . Early adverse experiences in schizophrenia and unipolar depression. J Nerv Ment Dis. 2009;197(1):65–68. 19155813 10.1097/NMD.0b013e3181925342

[34] Stilo SA , Di Forti M , Mondelli V , Falcone AM , Russo M , O'Connor J , et al. Social disadvantage: cause or consequence of impending psychosis? Schizophr Bull. 2013;39(6):1288–1295. 10.1093/schbul/sbs112 PMC3796070 23091267

[35] Trotta A , Di Forti M , Iyegbe C , Green P , Dazzan P , Mondelli V , et al. Familial risk and childhood adversity interplay in the onset of psychosis. BJPsych Open. 2015;1(1):6–13. 27703716 10.1192/bjpo.bp.115.000158 PMC4995579

[36] McGrath JJ , McLaughlin KA , Saha S , Aguilar-Gaxiola S , Al-Hamzawi A , Alonso J , et al. The association between childhood adversities and subsequent first onset of psychotic experiences: a cross-national analysis of 23 998 respondents from 17 countries. Psychol Med. 2017;47(7):1230–1245. 28065209 10.1017/S0033291716003263 PMC5590103

[37] Stilo SA , Gayer-Anderson C , Beards S , Hubbard K , Onyejiaka A , Keraite A , et al. Further evidence of a cumulative effect of social disadvantage on risk of psychosis. Psychol Med. 2017;47(5):913–924. 27916012 10.1017/S0033291716002993 PMC5341492

[38] Merikukka M , Ristikari T , Tuulio-Henriksson A , Gissler M , Laaksonen M . Childhood determinants for early psychiatric disability pension: a 10-year follow-up study of the 1987 Finnish Birth Cohort. Int J Soc Psychiatry. 2018(3):20764018806936. 10.1177/0020764018806936 30394811

[39] Zhang W , Zhu Y , Sun M , Guo R , Wu G , Wang Z , et al. Longitudinal trajectories of psychotic-like experiences and their relationship to emergent mental disorders among adolescents: a 3-year cohort study. J Clin Psychiatry. 2019;80(4). 10.4088/JCP.18m12437 31347795

[40] Shevlin M , McElroy E , Christoffersen MN , Elklit A , Hyland P , et al. Social, familial, and psychological risk factors for psychosis: a birth cohort study using the Danish Registry System. Psychosis. 2016 8(2):1–11. 10.1080/17522439.2015.1113306. 26786001

[41] Schiavone S , Colaianna M , Curtis L . Impact of early life stress on the pathogenesis of mental disorders: relation to brain oxidative stress. Curr Pharm Des. 2015;21:1404–1412. 25564385 10.2174/1381612821666150105143358

[42] Weitoft GR , Hjern A , Haglund B , Rosen M . Mortality, severe morbidity, and injury in children living with single parents in Sweden: a population-based study. Lancet. 2003;361(9354):289–295. 12559862 10.1016/S0140-6736(03)12324-0

[43] Waldron M , Grant JD , Bucholz KK , Lynskey MT , Slutske WS , Glowinski AL , et al. Parental separation and early substance involvement: results from children of alcoholic and cannabis dependent twins. Drug Alcohol Depend. 2014;134:78–84. 24120074 10.1016/j.drugalcdep.2013.09.010 PMC3908916

[44] Windle M , Windle RC . Parental divorce and family history of alcohol disorder: associations with young adults' alcohol problems, marijuana use, and interpersonal relations. Alcohol Clin Exp Res. 2018;42(6):1084–1095. 29693716 10.1111/acer.13638 PMC5984164

[45] Sterne JA , White IR , Carlin JB , Spratt M , Royston P , Kenward MG , et al. Multiple imputation for missing data in epidemiological and clinical research: potential and pitfalls. BMJ. 2009;338:b2393. 19564179 10.1136/bmj.b2393 PMC2714692

[46] Cohen GJ , Weitzman CC . Helping children and families deal with divorce and separation. Pediatrics. 2016;138(6):e20163020. 27940730 10.1542/peds.2016-3020

[47] Cuijpers P , Smit F , Unger F , Stikkelbroek Y , Ten Have M , de Graaf R . The disease burden of childhood adversities in adults: a population-based study. Child Abuse Negl. 2011;35(11):937–945. 22099144 10.1016/j.chiabu.2011.06.005

